# Viral codon usage and the virus-host interactions

**DOI:** 10.3389/fmicb.2025.1711603

**Published:** 2025-11-10

**Authors:** Thanyaporn Sirihongthong, Prasert Auewarakul

**Affiliations:** 1 Department of Microbiology, Faculty of Medicine Siriraj Hospital, Mahidol University, Bangkok, Thailand; 2 Emerging Infectious Diseases Research Unit, Research Department, Faculty of Medicine Siriraj Hospital, Mahidol University, Bangkok, Thailand

**Keywords:** viral codon usage, virus-host interactions, codon-specific translation, tRNA modification, wobble-base pairing, antiviral target

## Abstract

Codon usage pattern is a specific characteristic of each species as a result of evolution and interaction between genome composition and translational machinery. Species-specific optimal codon usage is a requirement for efficient expression in cells of that species. Viruses pose a curious situation where their genomes must interact with their hosts. Codon usage and genome composition of most viruses infecting eukaryotic hosts are markedly different from those of their hosts. How these viruses efficiently express their genes with non-optimal codon usage is not well understood. Some evidence suggests that they may manipulate host translational machinery to achieve this. On the other hand, host cells may launch innate antiviral defense to suppress expression of viral genes with non-optimal codon usage. Codon usages of viruses are more similar among viruses within the same genome type. This suggests that there may be common mechanisms driving codon usage of viruses within the same genome type. These interactions may contribute to host adaptation in inter-species transmission and viral emergence. However, direct adaptation to be more similar with host codon usage pattern is not always the case. Complex viral-host interaction may direct evolution of viral codon usage. More understanding in these interactions may provide new insight into the viral evolution and host adaptation and offer new possibilities in fighting against new and old viruses. Here we review various aspects of these interactions.

## Introduction

There are a total of 64 triplet codon combinations and only 20 proteinogenic amino acids in the standard codon table. An amino acid is therefore coded by either one, two, three, four, or six different codons. These iso-coding codons are degenerate at the third codon position in either C/U (for Phe, Tyr, His, Asn, Asp and Cys) and G/A (for Gln, Lys and Glu) pairs for the 2-box sets or three bases for the 3-box for Ile or all four bases for the 4-box sets (for Val, Pro, Thr, Ala and Gly), and the 6-box sets (for Leu, Ser and Arg) combine a 4-box set with a 2-box set to encode an amino acid (Table 1). These iso-coding or synonymous codons are not equally used in most genes and genomes. Each species prefers specific codon type resulting in species-specific codon usage bias (Grantham et al., 1980). The main forces shaping this codon usage bias are nucleotide composition, which is driven by mutational bias, selection pressures to enrich or avoid certain nucleotide composition, and selection pressure on translational efficiency (Sharp et al., 2010).

**Table 1:** Synonymous codons, numbers of tRNA isodecoder genes, wobble base pairing and tRNA modifications.

Amino acid	Codon^(a)^	Anticodon^(b)^	# tRNA genes^(c)^	Decoded by tRNA with 1^st^ anticodon position^(d)^	Modification that may affect wobble base pairing^(e)^
Two-box set with C- or U-ending					
Asparagine (Asn)	AA**C** AA**U**	**G**UU AUU	23 1	**G** to **C**	Guanosine modification by Queuine for Asn, Asp., His, and Tyr
Aspartic acid (Asp)	GA**C** GA**U**	**G**UC AUC	15 -		
Histidine (His)	CA**C** CA**U**	**G**UG AUG	10 -		
Tyrosine (Tyr)	UA**C** UA**U**	**G**UA AUA	12 1	**G** wobble to **U** or A to **U**	
Cysteine (Cys)	UG**C** UG**U**	**G**CA ACA	30 -		
Phenylalanine (Phe)	UU**C** UU**U**	**G**AA AAA	12 -		
Two-box set with G- or A-ending					
Lysine (Lys)	AA**G** AA**A**	**C**UU **U**UU	16 14	**C** to **G** **U** to **A** or **U** wobble to **G**	Modification of Uridine (thiolation and methylation)
Glutamic acid (Glu)	GA**G** GA**A**	**C**UC **U**UC	8 8		
Glutamine (Gln)	CA**G** CA**A**	**C**UG **U**UG	13 6		
Four-box set					
Alanine (Ala)	GC**C** GCU GCG GC**A**	GGC **A**GC CGC **U**GC	- 26 5 9	**A** → **I** to **C**, **A**, U A to U and C to G **U** to **A** or	Adenosine edited to Inosine, which pairs with C or A or U
Proline (Pro)	CC**C** CCU CCG CC**A**	GGG **A**GG CGG **U**GG	- 9 4 7		
Threonine (Thr)	AC**C** ACU ACG AC**A**	GGU **A**GU CGU **U**GU	- 9 5 6	**U** wobble to **C**, U, G	Modification of Uridine (thiolation and methylation)
	
Glycine (Gly)	GG**C** GGU GGG GG**A**	**G**CC ACC CCC **U**CC	14 - 7 9	**G** to **C** and **C** to **G** C to G and A to U **U** to **A** or **U** wobble to **C**, U, **G**	Modification of Uridine (thiolation and methylation)
Valine (Val)	GUC GUU GU**G** GU**A**	**G**AC **A**AC **C**AC **U**AC	- 10 15 5		
Six-box set					
Serine (Ser)	AG**C** AG**U**	**G**CU ACU	8 -	**G** to **C** **G** wobble to **U**	
	UC**C** UCU UCG UC**A**	GGA **A**GA CGA **U**GA	- 10 4 4	**A** → **I** to **C**, **A**, U A to U and C to G **U** to **A** or **U** wobble to **C**, U, G	Adenosine edited to Inosine, which pair with C or A or U Modification of Uridine (thiolation and methylation)
Arginine (Arg)	AG**G** AG**A**	**C**CU **U**CU	5 6	**C** to **G** **U** to **A** or **U** wobble to **G**	Modification of Uridine (thiolation and methylation)
	CG**C** CG**U** CG**G** CGA	GCG **A**CG **C**CG UCG	- 7 4 6	**A** → **I** to **C**, **A**, U **C** to **G** U to A or **U** wobble to **C**, **U**, **G** **A** to **U**	Adenosine edited to Inosine, which pair with C or A or U Modification of Uridine (thiolation and methylation)
Leucine (Leu)	UU**G** UU**A**	**C**AA **U**AA	7 4	**C** to **G** **U** to **A** or **U** wobble to **G**	Modification of Uridine (thiolation and methylation)
	CUC CUU CU**G** CU**A**	GAG AAG **C**AG **U**AG	- 9 9 3	**A** → **I** to C, **A**, U **C** to **G** **U** to **A** or **U** wobble to C, U, **G**	Adenosine edited to Inosine, which pair with C or A or U Modification of Uridine (thiolation and methylation)
Other set					
Isoleucine (Ile)	AU**C** AUU AU**A**	**G**AU **A**AU **U**AU	3 14 5	**G** to **C** **A** → **I** to **C**, **A**, U **U** to **A** or **U** wobble to **C**, U	Adenosine edited to Inosine, which pair with C or A or U Modification of Uridine to pseudouridine to prevent AUG mistranslation

Synonymous codons of each amino acid mainly classified to two-box, four-box and six-box sets. The nucleotide sequence of adenine, uridine, cytosine and guanine is represented as A, U, C and G, respectively.

(a) the most preferred codon usage of highly expressed human genes (bold blue letter-ending codons) and viral gene (bold red letter-ending codons) from Kazusa’s codon usage table of *Homo sapiens*, and comparison of codon usage between highly expressed human gene and HIV-1 *env* gene (Nakamura et al., 2000; Haas et al., 1996).

(b) tRNA anticodons with Watson-Crick base pairing and bold green letters are 1^st^ anticodon position of each tRNA.

(c) Number of tRNA genes from the hg19 reference human genome shown as # tRNA genes (Parisien et al., 2013).

(d) The bold color letters are represented the 1^st^ anticodon position of decoding tRNAs (green), the most preferred ending codon of highly expressed human genes (blue), the most preferred ending codon of viral gene (red), and the 1^st^ anticodon position of decoding tRNAs with wobble base pairing (purple).

(e) Modifications of tRNA that may affect wobble base pairing (Agris et al., 2018; Agris et al., 2007; Suzuki, 2021; Gu et al., 2014).

There is clear evidence that prokaryotes optimize their tRNA pools to match their codon usage (Kudla et al., 2009; Frumkin et al., 2018; Supek et al., 2010). Codon usage varies within a certain limit among genes within the same genome of the same species of prokaryotes, and the variation correlates well with the level of expression indicating a strong role of codon usage—tRNA pool matching (Sharp et al., 1988). Outliers of codon usage within the same genome is associated with horizontal gene transfer (Tuller et al., 2011). Viruses infecting prokaryotic hosts show similar genome composition and codon usage to those of their specific hosts (Simón et al., 2021). This is intuitively logical as the viruses would benefit from imitating their host genomes to avoid antiviral defense mechanisms targeting their genomes and to efficiently make use of host translational machinery. On the other hand, the role of codon usage is much less clear for eukaryotes. Although codon optimization to match that of highly expressed genes of the species enhance expression of foreign genes in eukaryote including human, global association between optimal codon usage and expression level is less clear (Ward et al., 2011; Rudolph et al., 2016; Sémon et al., 2005). Variations in levels of gene expression and tRNA isoacceptors in different types of cells and tissues add to the complexity (Pinkard et al., 2020; Dittmar et al., 2006). In accordance with this unclear role of codon usage in the hosts, viruses infecting eukaryotic hosts have codon usage pattern vastly different from those of their hosts (Gaunt and Digard, 2022). As human viruses are the most studied viruses, this review uses information on codon usage of human viruses to provide insights into codon usage of eukaryotic-host viruses and their interaction with hosts.

In terms of evolution, several studies reported no evidence of translational selective pressure on codon usage in humans (Rudolph et al., 2016; Callens et al., 2021; Kanaya et al., 2001). The codon usage is instead determined by local GC content that varies in clusters known as isochore or regions in chromosome banding, where GC-rich regions are lighter stained in G-banding and comprise euchromatin with more actively transcribed genes while AT-rich regions are darker and comprise less transcriptionally active heterochromatin (Sueoka and Kawanishi, 2000; Bernardi et al., 1985). Even though translational selective pressure may not drive human codon usage, the GC-rich euchromatin results in more favorable codon usage and hence support the organization of better codon usage with active genes. The cause of GC content variation and the isochore is still debated. Two explanations have been proposed: (1) more efficient mismatch repair in actively transcribed regions results in less mutations that lead to GC to AT conversion and hence higher GC content, and (2) actively transcribed regions in miosis leads to more frequent recombination that favors the transmission of GC-alleles over AT-alleles during meiosis and hence higher GC content (Duret and Galtier, 2009; Han et al., 2016; Supek and Lehner, 2015; Pouyet et al., 2017).

## Viral codon usage and genome

**Figure 1:**
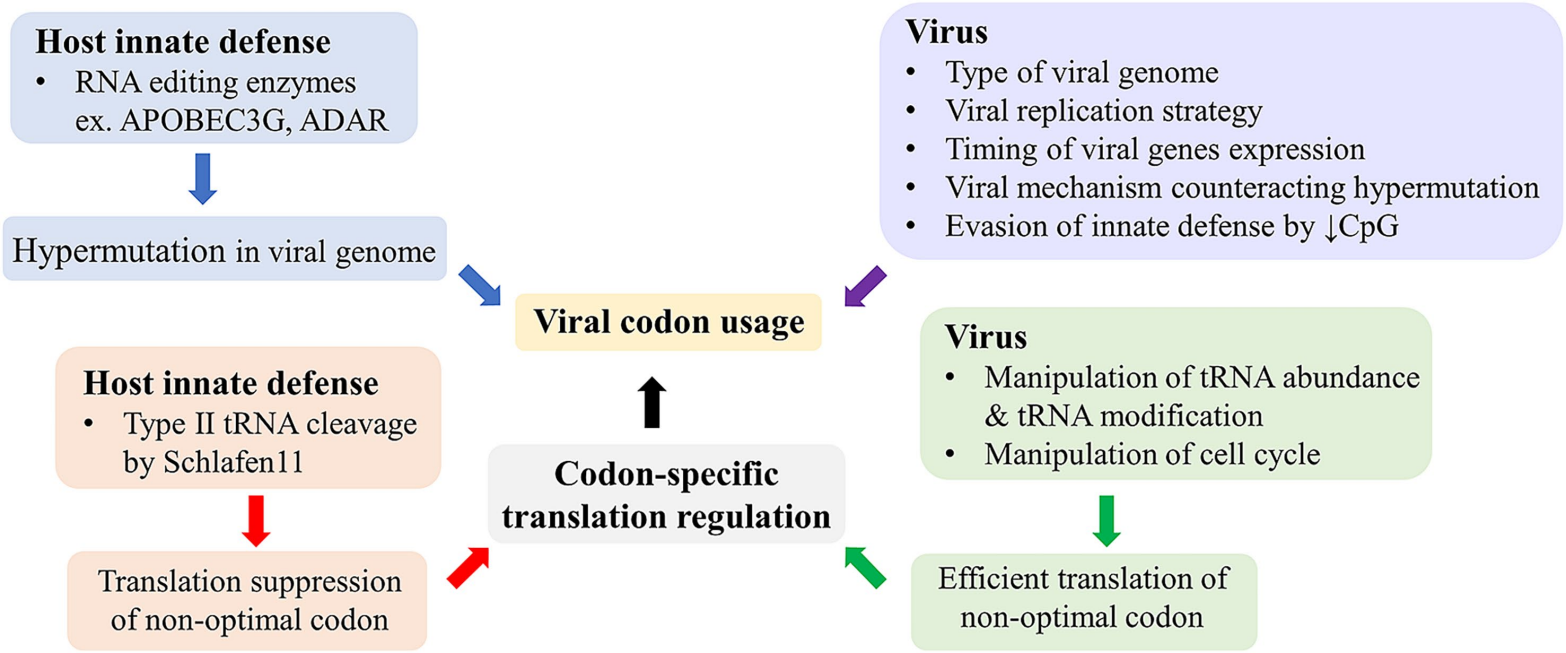
Virus-host interactions and the viral codon usage. Cellular innate defenses in the blue box can drive mutational bias and shape viral codon usage, whereas multiple viral factors in the purple box can influence viral codon usage via various mechanisms. On the other hand, host innate defenses in the red box can suppress viral gene expression in a codon-specific manner, while viruses have specific mechanisms, in the green box, to counteract these host innate defenses and enhance translation of their genes with non-optimal codon usage.

In general, codon usage patterns of human viruses are different from that of their host. There is an association between codon usage and genome types of human viruses (Sewatanon et al., 2007; Auewarakul, 2005; Phakaratsakul et al., 2018b; Phakaratsakul et al., 2018a). This may reflect the type of interaction of these viruses to their host. For DNA viruses, there is a difference between viruses with small and large genomes, where large viruses have more GC-rich genomes and more optimal codon usage in human context (Sewatanon et al., 2007). The reason for the difference in GC content between large and small viruses is unclear. Better mechanisms to maintain optimal codon usage made possible by their larger genome may be a reason. The presence of G-quadruplexes abundantly found in herpesvirus genomes, which may play some roles in genome stability and expression regulation (Zareie et al., 2024), may partly contribute to the high GC content of large DNA viruses. For RNA viruses, genome composition and codon usage of positive sense, negative sense, and double-stranded RNA are different (Auewarakul, 2005; Phakaratsakul et al., 2018b; Phakaratsakul et al., 2018a). Positive sense single-stranded RNA viruses tend to have higher GC content and more optimal codon usage than negative sense and double-stranded RNA viruses. Despite the difference, the overall codon usage pattern of most RNA viruses is generally more AU-rich and less optimal than most human genes (Auewarakul, 2005; Phakaratsakul et al., 2018b; Phakaratsakul et al., 2018a). In any case, codon usage of these viruses correlates well with GC content and genome composition. Genome composition is mainly driven by mutational bias where certain types of mutations are more frequent than others. The mutational bias may be caused by the nature of polymerase, composition of nucleotide pool at replication site, and cellular innate antiviral mechanisms driving hypermutation in viral genomes (Figure 1). Cytidine deamination by APOBEC3G and adenosine deamination by ADAR are among the mechanisms leading to hypermutation in viral genomes (Mangeat et al., 2003; Zhang et al., 2003; Suspène et al., 2011). The APOBEC3 enzyme family deaminates cytidine in single-stranded DNA and, to a lesser extent, single-stranded RNA leading to C-to-U and G-to-A hypermutation. APOBEC3G was shown to be a host defense against HIV-1 and other retroviruses, whereas other members of APOBEC3 family were shown to cause hypermutation in single and double stranded DNA viruses. Whether the enzymes can affect RNA viruses as well is still unclear (Jonathan and Ikeda, 2023). ADAR converts adenosine into inosine in RNA and causes A-to-G hypermutation. The enzyme has been shown to affect various types of RNA viruses (Piontkivska et al., 2021). While these enzymes are generally seen as host innate defenses to induce lethal mutations in viral genomes, the real interactions with viruses are complex and can result in both anti- and pro-viral effects (Phuphuakrat et al., 2008). In addition to mutational bias, viral genome composition can be affected by selective pressure on certain sequence characteristics. Genomes of many viruses were shown to contain a limited amount of CpG motif, which is a pathogen associated molecular pattern (Simmonds et al., 2013). This indicates selective pressure to evade cellular innate defense. In addition, dinucleotide bias and codon pair bias have been described as affecting codon usage in several viruses. A recent review covers these aspects of viral genome composition and codon usage (Simón et al., 2022). A summary of these host and viral factors shaping viral codon usage is shown in Figure 1.

## Host-specific adaptation of viral codon usage

Despite the global dissimilarity between viral and host codon usage, evidence for viral codon evolution toward host codon usage has been reported. Difference in codon usage was observed between avian and human influenza viruses. Furthermore, after interspecies jumping and a long-term evolution from zoonotic pandemic to seasonal influenza viruses, the viral codon usage shifted toward human virus pattern indicating an adaptation to the new host species (Wong et al., 2010). Whether this adaptation reduced the ability seasonal influenza viruses to jump back to avian species is unknown. It should be noted though that the adaptation does not necessarily result in codon usage that better matched with host codon usage. Host species-specific adaptation was also observed in human and animal rotaviruses (Wu et al., 2022; Hoxie and Dennehy, 2021). At a family level, various polyomaviruses with different specific animal host species showed different codon usage patterns (Cho et al., 2019). Codon usage of arthropod-borne flaviviruses can be grouped by their vector hosts (Lobo et al., 2009). On the other hand, codon usage of human and animal papillomaviruses does not show a host species-specific pattern (Van Doorslaer, 2013). It is unclear what determines the presence or absence of host species-specific viral codon adaptation.

Another plausible viral codon adaptation toward host is an adaptation to cell or tissue types. In addition to species-tropism, many viruses have clear cell or tissue-tropism. It was shown that there was a match between viral codon usage and preferred codon usage in target cell types for SARS-CoV2 (Hernandez-Alias et al., 2021; Zhou et al., 2022). Although there are house-keeping genes, which are commonly highly expressed in most types of cells, various cell types may have their own specific function and set of highly expressed genes not shared by others. It was shown that compositions of isoacceptor tRNA were different among cell types and organs, which matched with codon usage of genes of specific function in those cell types and organs (Pinkard et al., 2020; Dittmar et al., 2006). It should be noted that relative abundance of isoacceptor tRNA does not cover all the property of codon-specific translation efficiency, as tRNA modifications such as methylation and thiolation can influence wobble codon preference, and data on tRNA modification in different cell types are scarce.

## Modification of host translational machinery by viruses

It is intriguing how viral genes with non-optimal codon usage are efficiently expressed. A straightforward possibility is that viruses may alter host translational machinery to be more optimal for their codon usage (Figure 1). It is still debated whether viruses are capable of regulating tRNA abundance and other translational processes. HIV-1 and hepatitis E virus (HEV) were shown to manipulate tRNA pool in infected cells to enhance its translation (van Weringh et al., 2011; Ou et al., 2020). Vaccinia (VACV) and influenza viruses (IAV) were shown to select matched tRNA pool to polysome rather than alter the whole population of tRNA in the infected cells (Pavon-Eternod et al., 2013). On the other hand, it was clearly shown that hosts can target viral non-optimal codon usage of HIV-1 and some other viruses as an innate antiviral mechanism using Schlafen 11 protein, which represses viral protein translation by cleaving type II tRNA (Li et al., 2012; Li et al., 2018). More recent publications pointed to tRNA modifications as the main target for viral manipulation of the translational machinery. Chikungunya, Zika and SAR-CoV2 have been shown to activate modification of the wobble uridine of tRNA by the enzyme tRNA methyl transferase (TRMT)9A or B, which was important for efficient expression of viral genes (Jungfleisch et al., 2022; Eldin et al., 2024; Eldin and Briant, 2025; Muscolino and Díez, 2025). Another report showed that SAR-CoV2 protease cleaved and inhibited the activity of TRMT1, which methylates guanine at the position 26 of tRNA (Zhang et al., 2024). This downregulation of TRMT1 may suppress host protein expression and contribute to the viral pathogenesis.

## Functional implication of viral codon usage

Association between viral codon usage and replication strategy is another clue for its functional effect. Codon usages of plus sense single-stranded RNA viruses are more similar to human host than those of negative sense and double-stranded viruses (Phakaratsakul et al., 2018b; Phakaratsakul et al., 2018a). It was proposed that this may help translate the viral genome as it has to be translated immediately after entry into target cells, whereas negative sense and double-stranded RNA viruses make mRNA for translation after genome replication and there may be more time to modify host translation machinery. It was shown that codon usage of *tat* and *rev* genes of HIV-1 have more optimal codon than other genes (Phakaratsakul et al., 2018b; Phakaratsakul et al., 2018a). Similar explanation was proposed that these two genes are expressed early in the replication cycle and therefore need to be optimal for translation as the cellular translation machinery may not have yet been altered at this early phase of infection. Another curious finding was that free parvoviruses have codon usage distinctive from that of dependoviruses that need helper viruses to replicate (Sirihongthong et al., 2019). As they lack mechanisms to stimulate or alter cell cycle, free parvoviruses can replicate only in actively replicating cells (Bashir et al., 2001; Cotmore, S.F. et al., 2019). It is known that cell cycle-related human genes have a codon usage profile distinctive from highly expressed non-cell cycle-related genes (Gingold et al., 2014). Free parvoviruses use more AU-rich codons similar to cell cycle-related host genes suggesting that they are optimized for translation in actively replicating cells (Sirihongthong et al., 2019).

## Codon-specific translational regulation

Compatibility between codon occurrence in mRNA and the abundance of tRNA with matched anticodon provide optimal efficiency in translation, and mRNA with non-optimal codon usage cannot be efficiently translated because of the lack of matched tRNA. Adjustment of tRNA pool can therefore at least theoretically provide an additional level of expression regulation. There are multiple tRNA genes with similar anticodons, which are called isodecoder tRNA genes. And because there are multiple codons encoding for the same amino acid, there are multiple tRNA species, which may be different in anticodons, for translation of the same amino acid. These are called isoacceptor tRNA. Relative abundances of isoacceptor tRNAs as indicated by the numbers of tRNA genes partly correlate with the preferred codons of highly expressed human genes (Table 1). In addition, one tRNA species or one anticodon can recognize multiple codons through wobble base pairing (Agris et al., 2018). In wobble base pairing and anticodon - codon recognition; G can pair with U; I (inosine) can pair with U, A and C; and U can pair with all the bases or A and G (Table 1). The extent and preference of wobble base pairing is regulated by tRNA modifications (Agris et al., 2007). These post-transcriptional modifications of tRNA are highly complex and include methylation and thiolation at certain carbon positions of certain nucleotides in certain anticodon and non-anticodon positions of tRNA (Wang and Lin, 2023). There can be multiple modifications in a single tRNA molecule. These modifications regulate stability, translation efficiency and wobble base pairing of tRNA (Wang and Lin, 2023; Suzuki, 2021). Non-translation functions of tRNA and tRNA fragments have been also proposed (Zhang et al., 2023). Studies of these modifications are technically challenging and their regulation and roles in physiology and diseases are not well understood. Improved techniques have recently made it an area of intense investigation. In addition to the tRNA abundance and modification, codon-specific translational regulation can also be mediated by mRNA translation rate and codon-dependent mRNA stability (Presnyak et al., 2015).

Regulation of translation through codon usage and tRNA population can be at either transcriptional or post-transcriptional level. Transcriptional regulation controls abundance of tRNA isoacceptors, whereas post-transcriptional regulation affects their function. While the role of tRNA and codon-specific translational regulation on cell type- or tissue-specific expression control is still debated, cell cycle-associated fluctuation of tRNA pool is a well-known phenomenon (Aharon-Hefetz et al., 2020). Cell cycle-associated genes are more AT-rich (Frenkel-Morgenstern et al., 2012). This results in non-optimal codon usage, which does not allow efficient expression under normal conditions in non-replicating cells. These genes can only be efficiently expressed in cells during active cell cycle. It is still debated whether this is due to a regulatory mechanism specific to tRNA species required for AU-rich codons or just an increase in overall abundance of all tRNA surpassing a threshold for efficient expression of genes with non-optimal codon usage. The distinctive codon usage of cell cycle-related genes means that cancer cells may have tRNA pool and translational machinery conditioned differently from normal cells (Earnest-Noble et al., 2022). This may be a potential novel anticancer target. Various types of tRNA modifications and their enzymes have been shown to be associated with certain cancers and their prognosis. It was proposed that these can be used as biomarkers. Some RNA modification enzymes have been used for inhibitor screening. Some of the inhibitors have shown promising results in animal studies (Yuan et al., 2024).

Many viruses are known to interfere with cell cycle (Fan et al., 2018). Some viruses activate cell cycle to make cellular condition conducive to DNA synthesis and viral DNA replication. Some viruses stop cell cycle at the phase that is conducive to RNA and protein synthesis (Fan et al., 2018). These make the conditions in infected cells of many viruses optimal for expression of cell cycle-related genes and viral genes with similar codon usage. We have previously shown that although viral codon usage of human RNA viruses is different from the global codon usage of human, their codon usage is similar to a subset of human genes including cell cycle-related genes (Jitobaom et al., 2020). This may at least partially explain how viruses efficiently express their genes with non-optimal codon usage.

Codon-specific regulation by tRNA modification was proposed for a set of genes called MoTTs (modification tunable transcripts). These genes have unique codon usage and are mostly stress response genes (Endres et al., 2015; Gu et al., 2014). It was proposed that rapid changes in tRNA modification can swiftly alter expression levels of these genes in response to various stress signals without changes in mRNA and tRNA abundance, which may require more time. These MoTTs were mostly shown in yeast, and whether similar mechanisms exist in higher eukaryotes and humans await further studies.

## Innate defense targeting viral codon usage

Schlafen 11 is an interferon inducible innate antiviral protein. It was shown to inhibit HIV-1 and several other viruses in a codon-specific manner (Li et al., 2012). It binds to tRNA and was shown to prevent changes of tRNA repertoire induced by HIV-1 infection. It was later shown that Schlafen 11 and other proteins in the Schlafen family also inhibited other viruses (Jitobaom et al., 2023; Kim and Weitzman, 2022). The inhibitory mechanism was proposed to be cleavage of type II tRNA, which includes all tRNA of serine and leucine (Li et al., 2018). The type II tRNA differs from type I tRNA by their longer variable loop located between anticodon loop and T loop. This cleavage of type II tRNA was shown to be responsible for DNA damage-induced cell death (Li et al., 2018). Although all type II tRNAs are susceptible to the cleavage by Schlafen 11, some may be more susceptible than others leading to codon-specific inhibition. It was shown that tRNA-Leu-TAA was among those efficiently cleaved by Schlafen 11 and a reporter gene using only TTA for leucine was repressed by Schlafen 11, whereas another reporter gene using only CTT was not. This codon-specific antiviral mechanism is in concordance with the innate defense by APOBEC3G, which drives G-to-A hypermutation in HIV-1 genome resulting in A-rich genome and codon usage, which may be more effectively targeted by Schlafen 11 (Mangeat et al., 2003). HIV-1 evolved a specific mechanism to counteract the APOBEC3G function using *vif* gene (Mangeat et al., 2003). It can be therefore said that hosts and viruses fight to regulate codon-specific expression with either directly or indirectly counteracting mechanisms (Figure 1).

## Codon usage and translational regulation as a target for antiviral development

It is generally well accepted that codon usage patterns of most viruses do not match those of their hosts. However, this unmatched viral codon usage exists only when compared with average codon usage or codon usage of highly expressed genes of the hosts (Jitobaom et al., 2020). While the unmatched at the level of genome is consistent in most viruses, host codon usage is heterogenous among different genes or groups of genes within the same genome. We have previously shown that codon usages of human RNA viruses were actually similar to a group of human genes. Enrichment analysis identified among others cell cycle-related genes and DNA repair genes in those showing similar codon usage to those of viruses (Jitobaom et al., 2020). Previous reports also showed that cell cycle-related genes and non-cell cycle-related genes had different codon usage patterns, and that rapidly proliferating cells like cancer cells had translational condition that promoted translation of cell cycle-related genes (Aharon-Hefetz et al., 2020; Frenkel-Morgenstern et al., 2012; Earnest-Noble et al., 2022). This suggests the existence of some codon-specific translational control mechanisms, which may involve activation of tRNA transcription through RNA polymerase III (Pol III) promotor and tRNA modifications. The main mechanism controlling Pol III promotor is Maf1, which is a transcription repressor under the control of Akt kinase (Palian et al., 2014; Graczyk et al., 2018). And Akt is in turn controlled by various growth and metabolic signals including PI3K and PDK1 (Wu and Hu, 2010). These mechanisms are mainly controlled by cell cycle and metabolic regulation. Inhibitors of these signals may theoretically inhibit tRNA transcription and hinder translation of mRNA with non-optimal codon usage and hence viral protein synthesis. The inhibition should affect genes with non-optimal codon usage more than genes with optimal codon usage as they are more dependent on abundance of tRNA pool. In agreement with this hypothesis, knocking out Maf1 was shown to enhance replication of a fish virus (Kim et al., 2021). On the other hand, tRNA modification is very complex. There are many types of tRNA modifications, and it is unclear how those modifications affect codon-specific translation. The modifications of the most interest are those at the first anticodon position, which pair to the third wobble codon position. The modifications at this position previously shown to affect the choice of wobble base pairing are the replacement of guanosine by queuosine, thiolation and methylation of uridine (Sochacka et al., 2017; Müller et al., 2019; Laxman et al., 2013). A uridine methyltransferase enzyme, TRMT9B responsible for uridine modification at the wobble first anticodon position, was shown to be upregulated by chikungunya virus together with an increase in 5-methoxy-carbonyl-methyluridine (Jungfleisch et al., 2022). As the wobble choice can theoretically affect the efficiency of translation of AU-rich viral codons, agonists or antagonists of these tRNA modification may offer an approach to regulate viral gene expression in a codon-specific manner.

## Conclusion and future prospect

**Figure 2:**
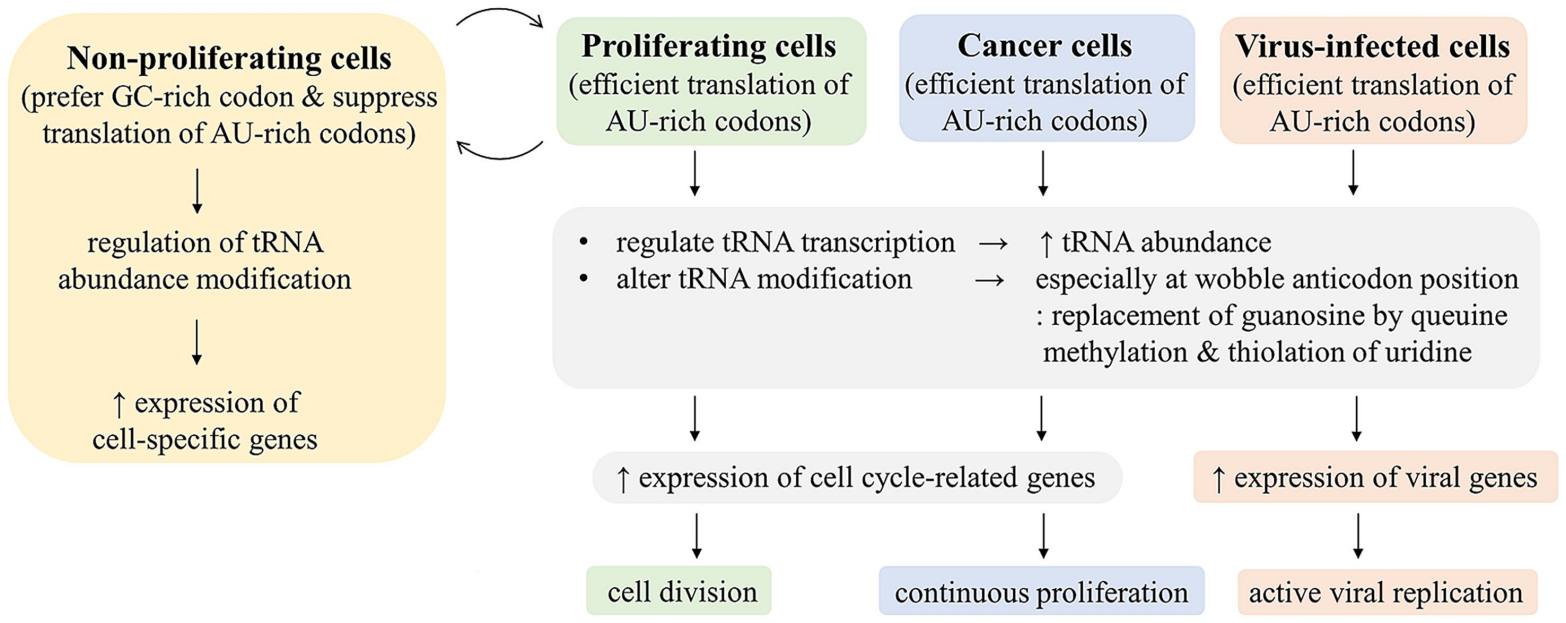
Cellular regulations to facilitate expression of genes with non-optimum codon usage. Most viral and cell cycle-related genes are more AT-rich and have less optimal codon usage than highly expressed human genes. In order to efficiently express these genes with poor codon usage, tRNA pool needs to be regulated for AU-rich codon translation in cancer, proliferating, and virus-infected cells by increasing tRNA abundance and tRNA modification, especially at the wobble anticodon position.

Previous studies on viral codon usage mostly aimed at understanding the viral evolution, especially host species adaptation. While this may offer some prediction of host adaptation, it does not provide further practical applications. More insights into the viral codon usage involve the role of host innate defenses and their interaction with viruses in shaping viral codon usage and the viral-host interaction to regulate codon-specific translation either suppression of viral gene translation by host or facilitation of viral gene translation by viruses (Figure 1). Codon usages of most viruses are AU-rich and non-optimal in human context, but they are somehow efficiently expressed in infected cells. The viral non-optimal codon usage shares similarity with that of cell cycle related genes, which are highly expressed in cancer cells. Although codon usage of human genes has been shown to be driven mainly by GC content and isochor, difference in codon usage among groups of genes and the association with gene function have been a subject of recent intense investigation, and it may provide a new approach for anticancer development (Earnest-Noble et al., 2022; Gillen et al., 2021; Meyer et al., 2021; Benisty et al., 2020). Cancer and viral infection may share some common mechanisms to facilitate efficient expression of these genes with non-optimal codon usage. The mechanisms may include regulations of tRNA abundance and modifications (Figure 2). These mechanisms are being targeted for anti-cancer development. It may also provide a new approach to anti-viral development. Recent evidence indicated that the enzymes TRMT9A and B, which methylate the wobble uridine of tRNA and were shown to be essential for efficient expression of a number of viruses (Jungfleisch et al., 2022; Eldin et al., 2024; Talló-Parra et al., 2022), may be a promising antiviral target.
